# RBPmap: a web server for mapping binding sites of RNA-binding proteins

**DOI:** 10.1093/nar/gku406

**Published:** 2014-05-14

**Authors:** Inbal Paz, Idit Kosti, Manuel Ares, Melissa Cline, Yael Mandel-Gutfreund

**Affiliations:** 1 Department of Biology, Technion - Israel Institute of Technology, Technion City, Haifa 32000, Israel; 2 Department of Molecular, Cellular and Developmental Biology, UCSC, Santa Cruz, CA, USA; 3 Center for Biomolecular Science & Engineering, UCSC, Santa Cruz, CA, USA

## Abstract

Regulation of gene expression is executed in many cases by RNA-binding proteins (RBPs) that bind to mRNAs as well as to non-coding RNAs. RBPs recognize their RNA target via specific binding sites on the RNA. Predicting the binding sites of RBPs is known to be a major challenge. We present a new webserver, RBPmap, freely accessible through the website http://rbpmap.technion.ac.il/ for accurate prediction and mapping of RBP binding sites. RBPmap has been developed specifically for mapping RBPs in human, mouse and *Drosophila melanogaster* genomes, though it supports other organisms too. RBPmap enables the users to select motifs from a large database of experimentally defined motifs. In addition, users can provide any motif of interest, given as either a consensus or a PSSM. The algorithm for mapping the motifs is based on a Weighted-Rank approach, which considers the clustering propensity of the binding sites and the overall tendency of regulatory regions to be conserved. In addition, RBPmap incorporates a position-specific background model, designed uniquely for different genomic regions, such as splice sites, 5’ and 3’ UTRs, non-coding RNA and intergenic regions. RBPmap was tested on high-throughput RNA-binding experiments and was proved to be highly accurate.

## INTRODUCTION

RNA-binding proteins (RBPs) play a central role in a variety of post-transcriptional regulatory processes, including splicing, mRNA localization, translation of mRNA as well as the regulation of non-coding RNA. Eukaryotic genomes contain hundreds of genes coding for RBPs, with diverse functions in co- and post-transcription regulation (1). While the binding preference (i.e. their specific binding motif) of the majority of RBPs is unknown, recent advances in *in-vivo* and *in-vitro* technologies have provided valuable resources for identifying the binding preferences of a large number of RBPs. RNAcompete was among the first high-throughput *in-vitro* methods for rapid and systematic analysis of the binding specificities of RBPs (2). Recently, Ray *et al.* have used thousands of short designed RNA oligos to determine the binding preferences of 207 different RBPs, mainly from human and *Drosophila melanogaster* (3). The detected binding preferences extracted from the latter experiments are listed in the Cis-BP-RNA website (http://cisbp-rna.ccbr.utoronto.ca). In recent years many large-scale assays have been developed to identify the targets of RBPs *in-vivo*. Among them are the Ribonucleoprotein immunoprecipitation (RIP) method (4) and the more advanced RIP-chip (5), as well as several cross-linking based methods such as CLIP (cross-linking immunoprecipitation) (6), CLIP-seq/HITS-CLIP (7), iCLIP (8) and PAR-CLIP (9). To date, several databases are available for browsing and extracting RBP binding results from *in-vivo* high-throughput binding experiments, such as CLIPZ (10) and doRINA (11). Following the extensive accumulation of experimental data for defining RBP targets, many new computational methods have been developed for *de-novo* motif predictions. Among them CMfinder (12), which uses a co-variation model for finding motifs in RNA sequences and MEMERIS (13), which is an extension of MEME (14) for finding enriched motifs in RNA sequences, incorporating RNA secondary structure information. Other *de-novo* motif discovery approaches such as AMADEUS (15), cERMIT (16) and DRIMust (17), which take advantage of the ranking of the target site for predicting enriched motifs in DNA and RNA sequences, are commonly employed for analyzing CLIP-data. 

Based on the accumulating data (from the aforementioned methods) on the binding preferences of RBPs, several databases for RBPs and RBP motifs have been generated. UTRdb and UTRsite are curated databases of experimentally validated functional motifs in 5’ and 3’ untranslated sequences of eukaryotic mRNAs, derived from several sources of primary data (18,19). Further, Cook *et al.* have generated a comprehensive database (RBPDB) of all RBPs, including their experimentally verified binding sites, when available (20). The RBPDB website allows users to scan a given sequence for potential RBP binding sites which are available in the database. In addition, several dedicated computational approaches have been developed to map binding motifs of RBPs, given a motif or a consensus sequence (19,21,22). We have developed the SFmap web service, specialized for mapping splicing factor (SF) binding sites on human genomic sequences given the experimentally defined binding motifs (23). SFma p search is based on our previously developed algorithm for predicting and mapping binding sites, which considers both the genomic environment of the motif and the evolutionary conservation of the binding site region (24). Specifically, SFmap utilizes a Weighted-Rank (WR) approach that considers the clustering propensity of SF binding sites. SFmap was tested and validated on high-throughput binding data for the NOVA and SRSF1 SFs, showing both high sensitivity and specificity. We have further validated SFmap predictions on CLIP data for the Polypyrimidine tract binding (PTB) protein and QKI, again demonstrating high sensitivity and specificity (25). SFmap predictions were further employed to derive the first splicing networks (24,25). Recently, Cereda *et al.* (26) have developed RNAmotifs for predicting *de-novo* clusters of RNA motifs that control alternative splicing. Zhang *et al.* have derived a hidden Markov model based algorithm named mCarts (27) to predict clustered functional RBP binding sites by effectively integrating the number and spacing of individual motif sites, their accessibility in local RNA secondary structures and cross-species conservation. The mCarts predictor was applied to two SFs, NOVA and MBNL, and demonstrated high reliable results which were validated experimentally.

Here we describe a new web service, RBPmap, which enables accurate prediction and mapping of binding sites of a wide range of different RBPs on any RNA sequence of interest, provided by the users. RBPmap has been developed specifically for mapping RBP binding sites in human, mouse and *D. melanogaster* genomes, though it supports other organisms too. RBPmap enables the users to select motifs from a database of 94 human/mouse and 51 *D. melanogaster* RBPs, whose experimentally defined motifs have been extracted from the literature as either a consensus motif or a Position Specific Scoring Matrix (PSSM). In addition, the user can provide any motif of interest given as either a consensus or a PSSM. RBPmap results are displayed in two web-based presentations, as a summary table of the predicted binding sites and in a visualized presentation of the binding sites mapped to the input sequence as custom tracks in the UCSC Genome Browser. RBPmap is freely accessible throughout the website http://rbpmap.technion.ac.il.

## RBPmap METHODOLOGY

**Figure 1.**
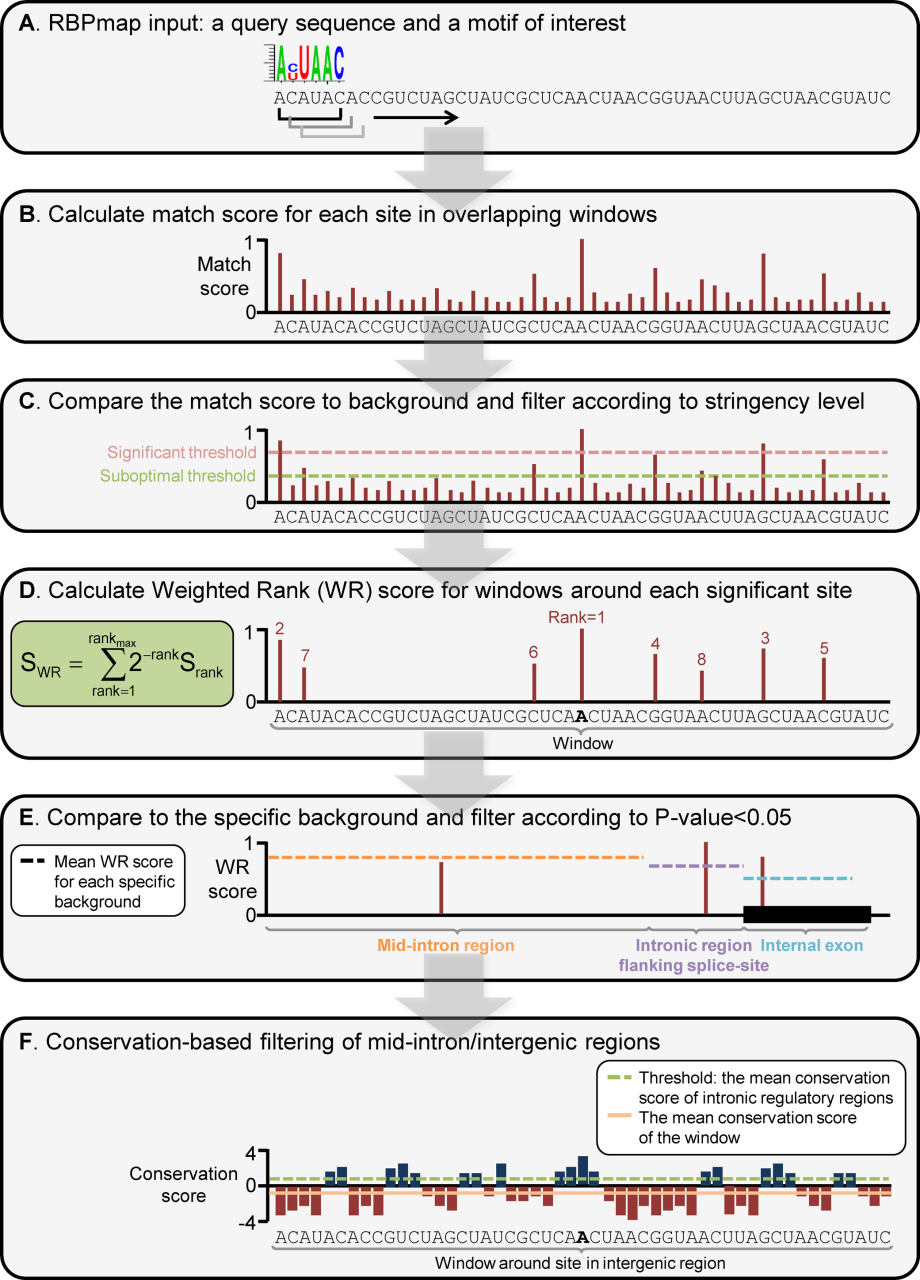
A pipeline summarizing RBPmap algorithm. (**A**) The mandatory input parameters for RBPmap run; a query sequence and a motif of interest to be mapped to the sequence. (**B**) A match score for the motif is calculated for each site in the query sequence, in overlapping windows of the motif size. (**C**) The match scores are compared to the average match score that is calculated for each motif in a background of randomly chosen regulatory regions. This step uses two different thresholds; a significant threshold for the anchor site (default *P*-value<0.005) and a suboptimal threshold for the secondary sites (default *P*-value<0.01) used to evaluate the clustering propensity. (**D**) A WR score is calculated for a window of 50 nts around each significant site. This score reflects the propensity of suboptimal sites to cluster around the significant site, weighted by their match score to the motif of interest. (**E**) To reduce false-positive predictions, the WR scores are compared to a region-specific background model that is generated independently per each motif for different genomic regions, removing non-significant results (*P*-value≥0.05). The figure exemplifies the procedure conducted for a query sequence spanning three different genomic regions (mid-intron, intronic region flanking a splice site and an internal exon). (**F**) Finally, a conservation-based filtering step is applied only to sites mapped to mid-intron/intergenic regions, filtering out sites which fall in non-conserved regions (below the average conservation level calculated for intronic regulatory regions).

The algorithm for mapping protein binding sites on the RNA sequences is based on our WR approach (24), previously exploited in the SFmap web server for mapping SF binding sites (23). The mapping algorithm considers the clustering propensity of the binding sites and the overall tendency of regulatory regions to be conserved (24). In RBPmap we have improved the algorithm by adding new features including the ability to map PSSM motifs, a conservation-based filtering to reduce the rate of false-positive predictions and a new background model which is specific to different genomic regions, namely intronic regions flanking the splice sites, internal exons, exons in 5’ and 3’ UTR regions, non-coding RNAs and mid-intron/intergenic regions (a detailed description of RBPmap algorithm is given in Supplementary file 1). A pipeline summarizing RBPmap algorithm is shown in Figure 1. Briefly, given an experimentally defined motif (provided as either a consensus sequence or a PSSM) and a query sequence (Figure 1A), RBPmap computes the match score for the motif per each position in the sequence in overlapping windows (Figure 1B). The match score is then compared to a background that is calculated specifically per each motif, filtering out all matches below a significant threshold (default *P*-value<0.005) (Figure 1C). At the next step, the WR function is employed to calculate the multiplicity score which reflects the propensity of suboptimal motifs (default *P*-value<0.01) to cluster around the significant motif in a window of 50 nts, weighted by their match to the motif of interest (24) (Figure 1D). Further, to reduce false-positive predictions, the final WR scores are compared to a background model that is calculated independently per each motif for the relevant genomic region. A Z-score is calculated for each WR score and coupled to a *P*-value, which represents the probability of obtaining a specific Z-score, considering a normal one-tailed distribution. RBPmap requires that the final WR score of a site will be significantly greater (with *P*-value<0.05) than the mean score calculated for the background, in order to consider this site as a predicted binding site (Figure 1E). The new position-specific background model provides more accurate and specific thresholds for the different regulatory regions on the RNA (see above). For sequences from genomes other than human, mouse or *Drosophila*, the WR scores are compared to a theoretical threshold instead of the genome-specific background model which cannot be obtained (see Supplementary file 1). This threshold is calculated for each motif separately, according to its length and complexity (23). At the last stage, we have added to the WR approach a conservation-based filtering, which exploits the tendency of regulatory regions to be evolutionary conserved. The conservation filter is optional and is applied only to sites that are mapped to mid-intron/intergenic regions on the query sequence. These positions are removed from the results if the mean conservation score of their environment is lower than the mean conservation score calculated for intronic regulatory regions (Figure 1F). For sequences from human and mouse, the conservation information is retrieved from the UCSC phyloP conservation table (28), based on the conservation of all placental mammals. For *Drosophila* sequences we use the phastCons insect conservation table (28). Both the position-specific background model and the conservation filtering are applied only for motifs which are searched in human, mouse or *Drosophila* sequences.

## RBPmap DESCRIPTION

### Input

**Figure 2.**
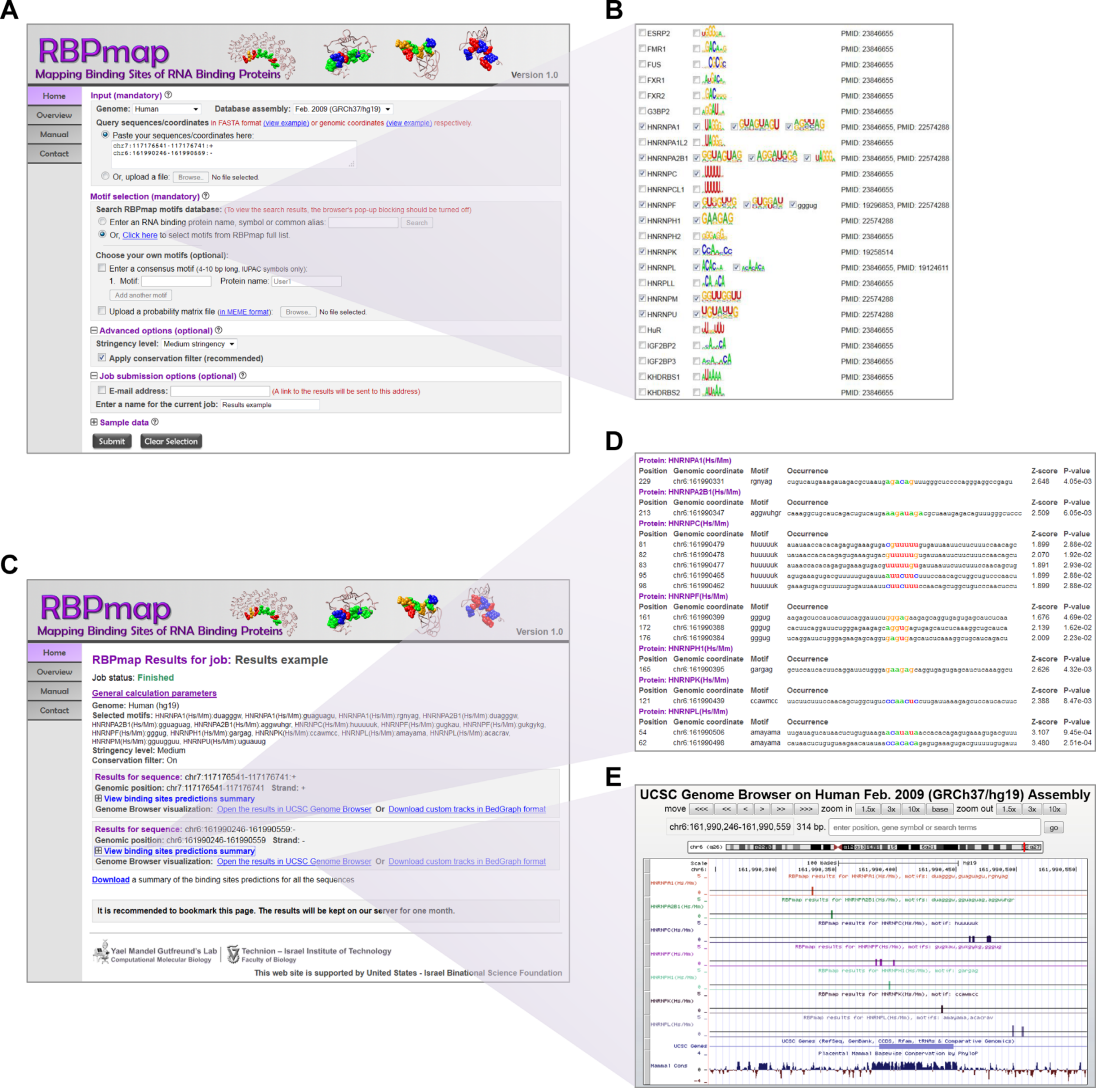
A view of RBPmap input and output pages. (**A**) An example of RBPmap home page demonstrating the mandatory input parameters needed for RBPmap run. (**B**) By clicking the link ‘Click here to select motifs from RBPmap full list’, a sorted list of all motifs in the RBPmap database is opened and the user is prompted to select the proteins/motifs of interest. (**C**) An example of RBPmap output page. In the example presented the job includes more than one query sequence. The results per each sequence are shown followed by a link to a text file summarizes the binding sites predictions for all the input sequences. (**D**) An example of the output summary of all predicted binding sites within one query sequence in a web-based presentation. The results are provided for each of the proteins selected by the user, where all the occurrences of motifs belonging to the same protein are listed together. (**E**) A visualized presentation of the predicted binding sites as custom tracks in the UCSC Genome Browser.

RBPmap is designed to predict and map RBP binding motifs in a query RNA sequence or a list of sequences. The server is designed for searching motifs in human, mouse and *Drosophila* genomes, for which it provides full functionality. Nevertheless, users can choose to search motifs of interest in other genomes. In the latter case, motifs will be searched without applying the position-specific background model and evolutionary conservation filtering (see below). The query sequences can be given in either FASTA format or provided as genomic coordinates (see Figure 2A). In case the sequences are provided in FASTA format, RBPmap employs the BLAT utility (29) to map each sequence to the chosen genome and retrieve its genomic coordinates (this option is restricted to human, mouse and *Drosophila* genomes). The minimal length for an individual sequence is 21 bp and the maximal length is 10,000 bp. However, long sequences can be divided and uploaded as separated sequences. The maximal number of entries per RBPmap run is 5,000. After uploading the input sequence/s the user is prompted to choose the motif/s of interest (Figure 2A). The user can select the motifs of interest from our RBPmap database, which currently includes 165 motifs of 145 different RBPs and/or enter custom motifs. The search engine of RBPmap enables entering a protein name, symbol or common alias. Alternatively, users can open the RBPmap list and select the motifs of interest manually (Figure 2B). Motifs, which are selected from the database or uploaded as custom motifs by the user, can be represented as either a PSSM in MEME format (14) or as a consensus motif using IUPAC symbols. Custom motifs will be predicted by the same algorithm used to map the motifs stored in our database. Notably, users can choose to combine within one run motifs from the database and custom motifs in all acceptable formats (see above).

In addition to the input motifs, among the advanced options, users can change the stringency level, employed by the WR algorithm to search for motifs matches (Figure 2A). The stringency can range between high, medium (the default) and low. The stringency level is defined by two thresholds (significant and suboptimal), used by RBPmap to calculate the WR function. For the high stringency level, the thresholds are at *P*-value_[significant]_<0.001 and *P*-value_[suboptimal]_<0.01. For the medium stringency level (default option), the thresholds are at *P*-value_[significant]_<0.005 and *P*-value_[suboptimal]_<0.01 and for the low stringency level, the thresholds are at *P*-value_[significant]_<0.01 and *P*-value_[suboptimal]_<0.02. These thresholds are calculated for each motif independently based on the genomic background. Another advanced parameter that can be controlled by the user is the conservation filtering (Figure 2A). It is applied as a default for intergenic regions only, but users can deliberately choose to skip this filtering. Conservation filtering is automatically ignored for sequences that do not align to the human, mouse or *Drosophila* genomes.

Finally, although not required, RBPmap supports including e-mail address to which the results will be automatically sent when the analysis is completed. This option is useful when submitting long jobs. The user is also capable of giving the job a specific informative name instead of the unique number it gets by default (Figure 2A).

### Output

RBPmap outputs the results for each query sequence in two web-based graphical presentations (Figure 2C), which are also available for download as text files. The first is a summary of the predicted binding sites within the query sequence, which is provided for each of the proteins selected by the user (Figure 2D). In case a selected RBP has more than one motif, the occurrences of all its ascribed motifs are listed together. The summary table includes the starting position of the binding site in the query sequence, its starting genomic coordinate, the mapped motif, the occurrence of the motif in the query sequence, highlighted in color and the statistical parameters for evaluating the significance of the matching. The statistical parameters include the Z-score, which measures the deviation of the site's WR score from the mean score calculated using the genome-specific background and the *P*-value of the Z-score, which represents the probability of obtaining a specific Z-score considering a normal one-tailed distribution. For sequences from genomes other than human, mouse *and Drosophila*, no genomic information is presented in the summary table and the statistical measures for evaluating the significance of prediction are the WR score and the theoretical threshold calculated for the corresponding motif. The summary table is presented on the website and can be downloaded as a text file. In case the job includes more than one query sequence, an additional text file, summarizes the binding sites predictions for all the sequences together, is available for download. An additional presentation of the results is provided as a visualized display of the binding sites mapped to the query sequence as custom tracks in the UCSC Genome Browser (Figure 2E). Each track represents a protein, and the predicted binding sites are displayed at their first genomic position. This presentation can be opened and displayed automatically in the UCSC Genome Browser and is also available for download as a text file in BedGraph format. Notably, for sequences from other genomes (excluding human, mouse and *Drosophila*) or in cases in which RBPmap could not map the query sequence to the requested genome with at least 95% identity, the output will not be displayed in the Genome Browser.

## RESULTS AND DISCUSSION

In recent years, an extensive number of *in-vivo* and *in-vitro* high-throughput techniques have been developed for detecting the targets of RBPs and extracting their binding preferences (4–9). Given the preferred binding sequences for a given RBP, several computational tools are currently available for mapping the motifs on a query sequence (18,20,22,29,30). These mapping algorithms rely on detecting homologous short sequences to the known motifs within the genomic region of interest, without considering context-dependent effects. Recently, we have developed SFmap (23) for mapping putative SF binding sites in the human genome. The great advantage of SFmap, which implements the COS(WR) algorithm (24), is that it considers not only the homology of the sequence to the known motif but it also takes into account the properties of the motif environment, including the clustering propensity of binding sites and the overall tendency of regulatory regions to be conserved. These additional features allow SFmap to be highly accurate with a relatively low false detection rate (24,25). Given the great advance in the experimental high-throughput technologies and the accumulation of data on the binding preferences of many RBPs of diverse functions, we have now developed RBPmap for detecting the binding motifs of any RBP which can be selected from the database of experimentally defined binding motifs from *in-vivo* (e.g. 9,31) or *in-vitro* (3) studies or otherwise provided by the user. To fit the mapping algorithm for searching motifs of any RBP of interest, we have constructed a new genomic background model that generates a unique region-specific threshold per each motif. The background model captures the genomic properties of the different regulatory regions of the query sequence, such as splice sites, 5’ and 3’ UTRs, non-coding RNAs and mid intron/intergenic regions, requiring the predicted motif to have a score which is significantly higher than the average score for a motif within the given region. To validate RBPmap predictions and show its added value in filtering out false-positive predictions, we have tested it on 10 different datasets of high-throughput RNA-binding data extracted from CLIP experiments, for which information on the binding affinity of the RBP to the sequence could be deduced from the data and the defined binding motifs were available from our dataset. Finally, the test was performed for 10 different RBPs including five hnRNPs (32), PTB (33), both generated using hits-clip experiment, TDP43 from I-CLIP (34) and QKI (9), HuR (9) and PUM2 (9) from PAR-CLIP. From each dataset we extracted the 1000 top ranked CLIP sequences (strong binders) and the 1000 bottom ranked set of sequences (weak binders) (excluding hnRNPA1, in which we extracted only 500 top-ranked and 500 bottom-ranked sequences, which were restricted by the size of the dataset). For the hnRNPs and PTB, the ranked data was obtained directly from the original studies (32,33). The ranked data for TDB43 was extracted from the doRiNA database (11). The PAR-CLIP data was sorted using the PARalyzer tool (35), employing the standard protocol for ranking PAR-CLIP data based on the percent of C to T conversion centered at the anchor site and further normalized for RNA abundance. We then employed RBPmap to map the known binding motifs to the given sequences and performed the Fisher's exact test to evaluate the statistical significance of the enriched detected motifs in the set of strong binders relative to the motifs detected in the sequences at the bottom of the ranked list (as detailed in Supplementary Table S1). As shown in Supplementary Table S1 (column ‘WR score with conservation’), in all cases tested we have detected a significant enrichment of the mapped motifs in the set of the strong binders (ranked highest in the CLIP experiments) with highly significant p-values, ranging from 6.56e^−9^ to 3.97e^−207^ and an average sensitivity and specificity of 0.61 (±0.18) and 0.74 (±0.11), respectively. Since, to our knowledge, there are no other web services available to which we can compare the performance of RBPmap, we have conducted a comparative analysis between the results obtained by RBPmap, employing the WR algorithm (with and without the conservation filtering) and the results of RBPmap, based simply on the match score of the motif. As shown in Table S1, when comparing the results in the column ‘Match score’ to the results in the column ‘WR score – no conservation’, in seven of the 10 experiments, the WR approach significantly improved the results. Furthermore, when adding the conservation filter (column ‘WR score - with conservation’ in Table S1), in all the experiments, except for hnRNPU, we obtained a significant improvement in the *P*-value compared to the results obtained using the match score only. Notably, while in some cases the overall *P*-value did not change radically, adding the conservation filtering substantially reduced the number of false positives for all RBPs, resulting in significantly higher specificity values. Overall, these results strongly demonstrate the strength of RBPmap to identify functional RBP binding sites with relatively high sensitivity and specificity.

Taken together, RBPmap provides the search of a comprehensive dataset of experimentally defined motifs of a diverge set of RBPs in the human, mouse and *Drosophila* genomes and in addition allows the users to search any motif of interest in any genome. The strength of the algorithmic approach, employed by RBPmap for accurate mapping of RBP motifs, lies in the fact that it takes into consideration information from the sequence environment considering the clustering propensity of protein binding sites. Furthermore, RBPmap uses a region-specific background model for adapting the motif-specific thresholds, used by the algorithm for removing noise, to the precise genomic content. In addition, given the well-established notion that functional motifs tend to fall within evolutionary conserved region, RBPmap uses a conservation-based filtering mechanism to remove motifs mapped to non-conserved intergenic sites. Nevertheless, to allow the identification of species-specific binding sites within these regions, RBPmap enables the user to deliberately avoid the conservation filtering. Finally, by adopting a content-dependent mapping approach, RBPmap can identify functional binding sites of RBPs on RNA sequences with a relatively low false-positive detection rate. Notably, while we believe RBPmap is a highly useful tool to direct researcher to sequences that can potentially target the RBPs of interest, clearly an experimental follow-up will be required to confirm these predictions.

## SUPPLEMENTARY DATA

Supplementary Data are available at NAR Online.

Supplementary Data
