# Supplementary material for: RBPmap: a web server for mapping binding sites of RNA-binding proteins
Source: Nucleic Acids Res. 2014 May 14;42(Web Server issue):W361–7. doi: 10.1093/nar/gku406 (PMC4086114; doi:10.1093/nar/gku406)
Supplement: Supplementary Data [file supp_42_W1_W361__index.html]

Supplementary Data 

# RBPmap: a web server for mapping binding sites of RNA-binding proteins

## Supplementary Data

**Files in this Data Supplement:**

- SUPPLEMENTARY DATA
- SUPPLEMENTARY DATA
