# Supplementary material for: RBPmap: a web server for mapping binding sites of RNA-binding proteins
Source: Nucleic Acids Res. 2014 May 14;42(Web Server issue):W361–7. doi: 10.1093/nar/gku406 (PMC4086114; doi:10.1093/nar/gku406)
Supplement: Supplementary Data [file supp_gku406_nar-00467-web-b-2014-File004.pdf]

Supplementary file 1

RBPmap algorithm

The algorithm for mapping protein binding sites on the RNA sequences is based on our Weighted-Rank (WR) approach (1), previously exploited in the SFmap web-server for mapping splicing factor binding sites (2). The mapping algorithm considers the clustering propensity of the binding sites and the overall tendency of regulatory regions to be conserved (1).

Processing the query sequence (for human, mouse and Drosophila genomes)
The mandatory input parameters for RBPmap are a query sequence and at least one motif of interest to be mapped to the sequence. The query sequence can be provided as a sequence in FASTA format or as genomic coordinates (for human, mouse and *Drosophila melanogaster* genomes). In case the query sequence is provided in FASTA format, RBPmap uses the BLAT (3) utility to map the sequence to the chosen genome and retrieve the genomic coordinates. The sequence is then expanded by 25nts upstream and downstream to include the sequence environment in the WR calculation (see below).

Further, the sequence is mapped to the genome and categorized to one of five different genomic regions: intronic regions flanking the splice sites (80nts long), internal exons, exons in 5' and 3' UTR regions, non-coding RNA and mid-intron/intergenic regions. The category of the sequence is further used to choose the region-specific background model (see below).

Calculating a match score for the motif
Given a query sequence and a motif (defined as either a consensus sequence or a PSSM, selected from RBPmap database or provided by the user), a match score for the motif is calculated for each k-mer of the motif size in the query sequence, in overlapping windows. The match score $S_{consensus}$, for motif provided as a consensus sequence, is defined as following:

$$S_{concensus} = \frac{L - H}{L}$$

Where *L* is the motif length and *H* is the Hamming distance between each k-mer and the motif.
The match score S_{pssm}, for a PSSM (Position Specific Scoring Matrix), is defined as following:

$$S_{pssm} = \sum_{i=1}^{L} f_{N_i}$$

Where L is the motif length, N_i is the specific nucleotide in position i and f_{Ni} is the frequency of the nucleotide as defined in the PSSM.
The values of both match scores range between 0 and 1, increasing as the distance between the motif and the k-mer decreases.

Comparing the match scores to a background model

For defining a significant match, the match scores of all the sites in the query sequence are compared to the mean match score for the motif calculated for a background of randomly chosen regulatory regions (composed of exonic and intronic regions around splice-sites and exons in UTR regions). Z-scores are calculated and coupled to a P-value, which represents the probability of obtaining a specific Z-score considering a normal one-tailed distribution. The sites are filtered according to two thresholds (set by the user as the *stringency level* parameter): *significant threshold* (default P-value<0.005) and *suboptimal threshold* (default P-value<0.01). The significant threshold is used to define the putative binding site and the suboptimal threshold, which is less stringent, filters the sites that are clustered around the putative binding site and will be considered in the Weighted Rank (WR) score (see below).

Calculating a Weighted Rank (WR) score for windows around each putative binding site

In order to calculate a multiplicity score, which reflects the propensity of suboptimal motifs to cluster around the significant motif, a Weighted Rank (WR) function is employed (1). The WR score is calculated for each candidate significant site, by summing up all suboptimal match scores within a window of 50nts around the site (25nts of each side), weighted by their match to the motif of interest (the significant site is ranked first). The WR score S_{WR} is defined as following:

$$S_{WR} = \sum_{rank=1}^{rank_{max}} 2^{-rank} * S_{rank}$$

Where $rank_{max}$ is the number of suboptimal sites within the 50nts window and S_{rank} is the match score of each ranked suboptimal site.

Comparing the WR scores to a region-specific background model

In order to reduce the false positive predictions, the final WR scores are compared to a background model, which is calculated independently for 5 different genomic regions (see above). The WR score of each putative binding site is compared to the mean WR score of its pre-defined genomic region. Z-scores are calculated and coupled to a P-value, which represents the probability of obtaining a specific Z-score considering a normal one-tailed distribution. The sites are reported as predicted binding sites if their P-

value<0.05. The Z-score and P-value of the predicted binding sites are reported in the output of RBPmap.

Conservation-based filtering

The conservation-based filtering is optional and can be applied only to binding sites that are mapped to intronic/intergenic regions. It is based on the tendency of regulatory regions to be evolutionary conserved. These sites are removed from the final results if the mean conservation score calculated for their window is lower than the mean conservation score calculated for intronic regulatory regions. For sequences from human and mouse, the conservation information is retrieved from the UCSC phyloP conservation table (4), based on the conservation of all placental mammals. For *Drosophila* sequences we use the phastCons insect conservation table (4). Conservation filtering can be applied only for input sequences from human mouse or *Drosophila*.

RBPmap calculation for genomes other than human, mouse or Drosophila

In case the query sequence comes from other organism than human, mouse or *Drosophila melanogaster*, RBPmap cannot use any background genomic information. Thus, all the above steps are performed, except for the comparison to the genome-specific background model and the conservation-based filtering. Nevertheless, to reduce the false-positive predictions, the WR scores are compared to a theoretical threshold, calculated for each motif, based on the motif length and complexity (2). In such cases, the reported output of RBPmap includes the final WR scores (‘Score’) and the theoretical threshold for each motif (‘Cutoff’).

References

1. Akerman, M., David-Eden, H., Pinter, R.Y. and Mandel-Gutfreund, Y. (2009) A computational approach for genome-wide mapping of splicing factor binding sites. *Genome Biol.*, **10**, R30, doi:10.1186/gb-2009-10-3-r30, http://www.ncbi.nlm.nih.gov/pmc/articles/pmid/19296853/.

2. Paz, I., Akerman, M., Dror, I., Kosti, I. and Mandel-Gutfreund, Y. (2010) SFmap: a web server for motif analysis and prediction of splicing factor binding sites. *Nucleic Acids Res.*, **38**, W281–285, doi:10.1093/nar/gkq444, http://www.ncbi.nlm.nih.gov/pmc/articles/PMC2896136/.

3. Kent, W.J. (2002) BLAT—The BLAST-Like Alignment Tool. *Genome Res.*, **12**, 656–664, doi:10.1101/gr.229202, http://www.ncbi.nlm.nih.gov/pmc/articles/PMC187518/.

4. Siepel, A., Bejerano, G., Pedersen, J.S., Hinrichs, A.S., Hou, M., Rosenbloom, K., Clawson, H., Spieth, J., Hillier, L.W., Richards, S., et al. (2005) Evolutionarily conserved elements in vertebrate, insect, worm, and yeast genomes. *Genome Res.*, **15**, 1034–1050, doi:10.1101/gr.3715005, http://www.ncbi.nlm.nih.gov/pmc/articles/PMC1182216/.
