# Supplementary material for: RBPmap: a web server for mapping binding sites of RNA-binding proteins
Source: Nucleic Acids Res. 2014 May 14;42(Web Server issue):W361–7. doi: 10.1093/nar/gku406 (PMC4086114; doi:10.1093/nar/gku406)
Supplement: Supplementary Data [file supp_gku406_nar-00467-web-b-2014-File005.pdf]

Table S1: Validating RBPmap on high throughput binding experiments

			Match score		WR score no conservation		WR score with conservation	
Protein	Motif(s)	Ref. to dataset	Testing results*	Measures of performance$	Testing results*	Measures of performance$	Testing results*	Measures of performance$
HnRNPA1	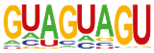 (1)<br>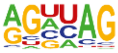 (1)<br>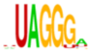 (2)	(1)	TP = 497<br>TN = 25<br>FN = 3<br>FP = 475	SN = 0.994<br>SP = 0.050<br>PV = $1.08e^{-5}$	TP = 441<br>TN = 177<br>FN = 59<br>FP = 323	SN = 0.441<br>SP = 0.177<br>PV = $3.80e^{-19}$	TP = 323<br>TN = 340<br>FN = 177<br>FP = 160	SN = 0.646<br>SP = 0.680<br>PV = $2.21e^{-25}$
HnRNPF	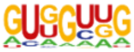 (1)<br>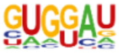 (1)<br>GGGUG (3)	(1)	TP = 925<br>TN = 266<br>FN = 75<br>FP = 734	SN = 0.925<br>SP = 0.266<br>PV = $2.84e^{-31}$	TP = 538<br>TN = 692<br>FN = 462<br>FP = 308	SN = 0.538<br>SP = 0.692<br>PV = $1.04e^{-25}$	TP = 444<br>TN = 830<br>FN = 556<br>FP = 170	SN = 0.444<br>SP = 0.830<br>PV = $2.63e^{-41}$
HnRNPH	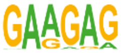 (1)	(1)	TP = 991<br>TN = 252<br>FN = 9<br>FP = 748	SN = 0.991<br>SP = 0.252<br>PV = $1.62e^{-70}$	TP = 956<br>TN = 352<br>FN = 44<br>FP = 648	SN = 0.956<br>SP = 0.352<br>PV = $5.75e^{-74}$	TP = 690<br>TN = 765<br>FN = 310<br>FP = 235	SN = 0.690<br>SP = 0.765<br>PV = $5.97e^{-96}$
HnRNPM	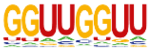 (1)	(1)	TP = 954<br>TN = 508<br>FN = 46<br>FP = 492	SN = 0.954<br>SP = 0.508<br>PV = $2.23e^{-132}$	TP = 879<br>TN = 645<br>FN = 121<br>FP = 355	SN = 0.879<br>SP = 0.645<br>PV = $2.76e^{-137}$	TP = 648<br>TN = 825<br>FN = 352<br>FP = 175	SN = 0.648<br>SP = 0.825<br>PV = $2.92e^{-107}$
HnRNPU	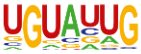 (1)	(1)	TP = 713<br>TN = 500<br>FN = 287<br>FP = 500	SN = 0.713<br>SP = 0.500<br>PV = $9.27e^{-23}$	TP = 381<br>TN = 793<br>FN = 619<br>FP = 207	SN = 0.381<br>SP = 0.793<br>PV = $6.82e^{-18}$	TP = 291<br>TN = 876<br>FN = 709<br>FP = 124	SN = 0.291<br>SP = 0.876<br>PV = $1.05e^{-20}$
HuR	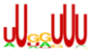 (2)	(4)	TP = 913<br>TN = 72<br>FN = 87<br>FP = 928	SN = 0.913<br>SP = 0.072<br>PV = $9.07e^{-1}$	TP = 860<br>TN = 162<br>FN = 140<br>FP = 838	SN = 0.860<br>SP = 0.162<br>PV = $9.48e^{-2}$	TP = 829<br>TN = 585<br>FN = 171<br>FP = 415	SN = 0.829<br>SP = 0.585<br>PV = $1.57e^{-84}$
PTB	CUCUCU (3)<br>UCUU (3)	(5)	TP = 999<br>TN = 0<br>FN = 1<br>FP = 1000	SN = 0.999<br>SP = 0<br>PV = 1	TP = 948<br>TN = 235<br>FN = 52<br>FP = 765	SN = 0.948<br>SP = 0.235<br>PV = $1.72e^{-33}$	TP = 503<br>TN = 624<br>FN = 497<br>FP = 376	SN = 0.503<br>SP = 0.624<br>PV = $6.56e^{-9}$
PUM2	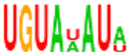 (4)	(4)	TP = 995<br>TN = 383<br>FN = 5<br>FP = 617	SN = 0.995<br>SP = 0.383<br>PV = $4.89e^{-126}$	TP = 995<br>TN = 683<br>FN = 45<br>FP = 317	SN = 0.995<br>SP = 0.683<br>PV = $2.48e^{-220}$	TP = 927<br>TN = 708<br>FN = 730<br>FP = 292	SN = 0.927<br>SP = 0.708<br>PV = $3.97e^{-207}$
QK1	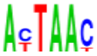 (4)	(4)	TP = 986<br>TN = 517<br>FN = 14<br>FP = 483	SN = 0.986<br>SP = 0.517<br>PV = $1.67e^{-171}$	TP = 876<br>TN = 865<br>FN = 124<br>FP = 135	SN = 0.876<br>SP = 0.865<br>PV = $3.70e^{-269}$	TP = 599<br>TN = 881<br>FN = 401<br>FP = 119	SN = 0.599<br>SP = 0.881<br>PV = $4.67e^{-118}$
TARDBP (TDP43)	UGUGUG (6)	(6)	TP = 1000<br>TN = 0<br>FN = 0<br>FP = 1000	SN = 1<br>SP = 0<br>PV = 1	TP = 968<br>TN = 339<br>FN = 32<br>FP = 661	SN = 0.968<br>SP = 0.339<br>PV = $9.16e^{-79}$	TP = 517<br>TN = 670<br>FN = 483<br>FP = 330	SN = 0.517<br>SP = 0.670<br>PV = $1.53e^{-17}$

* Performance was evaluated by counting the number of sequences in the target set in which the RBP has at least one significant hit, defined as True Positives (TP); number of sequences in the target set with no hits, defined as False Negatives (FN); number of sequences in the background set in which the RBP had at least one significant hit, defined as False Positives (FP); number of sequences in the background set in which the RBP had no hits, defined as True Negatives (TN).

$ Sensitivity (SN) and specificity (SP) were calculated as follows:

$$Sensitivity = \frac{TP}{TP+FN}$$

$$Specificity = \frac{TN}{TN+FP}$$

P-values (PV) were calculated using Fisher's exact test (one-tailed).

References

1. Huelga, S.C., Vu, A.Q., Arnold, J.D., Liang, T.Y., Liu, P.P., Yan, B.Y., Donohue, J.P., Shiue, L., Hoon, S., Brenner, S., et al. (2012) Integrative Genome-wide Analysis Reveals Cooperative Regulation of Alternative Splicing by hnRNP Proteins. *Cell Reports*, **1**, 167–178, doi:10.1016/j.celrep.2012.02.001, http://www.ncbi.nlm.nih.gov/pmc/articles/PMC3345519/.

2. Ray, D., Kazan, H., Cook, K.B., Weirauch, M.T., Najafabadi, H.S., Li, X., Gueroussov, S., Albu, M., Zheng, H., Yang, A., et al. (2013) A compendium of RNA-binding motifs for decoding gene regulation. *Nature*, **499**, 172–177, doi:10.1038/nature12311, http://www.ncbi.nlm.nih.gov/pubmed/23846655.

3. Akerman, M., David-Eden, H., Pinter, R.Y. and Mandel-Gutfreund, Y. (2009) A computational approach for genome-wide mapping of splicing factor binding sites. *Genome Biol.*, **10**, R30, doi:10.1186/gb-2009-10-3-r30, http://www.ncbi.nlm.nih.gov/pmc/articles/pmid/19296853/.

4. Hafner, M., Landthaler, M., Burger, L., Khorshid, M., Hausser, J., Berninger, P., Rothballer, A., Ascano, M., Jr, Jungkamp, A.-C., Munschauer, M., et al. (2010) Transcriptome-wide identification of RNA-binding protein and microRNA target sites by PAR-CLIP. *Cell*, **141**, 129–141, doi:10.1016/j.cell.2010.03.009, http://www.ncbi.nlm.nih.gov/pmc/articles/PMC2861495/.

5. Xue, Y., Zhou, Y., Wu, T., Zhu, T., Ji, X., Kwon, Y.-S., Zhang, C., Yeo, G., Black, D.L., Sun, H., et al. (2009) Genome-wide analysis of PTB-RNA interactions reveals a strategy used by the general splicing repressor to modulate exon inclusion or skipping. *Mol. Cell*, **36**, 996–1006, doi:10.1016/j.molcel.2009.12.003, http://www.ncbi.nlm.nih.gov/pmc/articles/PMC2807993/.

6. Tollervey, J.R., Curk, T., Rogelj, B., Briese, M., Cereda, M., Kayikci, M., König, J., Hortobágyi, T., Nishimura, A.L., Župunski, V., et al. (2011) Characterizing the RNA targets and position-dependent splicing regulation by TDP-43. *Nat Neurosci*, **14**, 452–458, doi:10.1038/nn.2778, http://www.ncbi.nlm.nih.gov/pmc/articles/PMC3108889/.
